# A Platform for Co-Culture of Primary Human Colonic Epithelium With Anaerobic Probiotic Bacteria

**DOI:** 10.3389/fbioe.2022.890396

**Published:** 2022-06-08

**Authors:** Raehyun Kim, Yuli Wang, Christopher E. Sims, Nancy L. Allbritton

**Affiliations:** ^1^ Department of Biological and Chemical Engineering, Hongik University, Sejong, South Korea; ^2^ Department of Bioengineering, University of Washington, Seattle, WA, United States

**Keywords:** oxygen gradient, probiotics, butyrate, gut microbiome, bacterial co-culture, intestine, organ on a chip

## Abstract

An *in vitro* platform was designed and optimized for the co-culture of probiotic anaerobic bacteria with a primary human colonic epithelium having a goal of assessing the anti-inflammatory impact of the probiotic bacteria. The device maintained a luminal O_2_ concentration at <1% while also supporting an oxygenated basal compartment at 10% for at least 72 h. Measurement of the transepithelial resistance of a confluent colonic epithelium showed high monolayer integrity while fluorescence assays demonstrated that the monolayer was comprised primarily of goblet cells and colonocytes, the two major differentiated cell subtypes of the colonic epithelium. High monolayer barrier function and viability were maintained during co-culture of the epithelium with the probiotic obligate anaerobe *Anaerobutyricum hallii* (*A. hallii*). Importantly the device supported a static co-culture of microbes and colonic epithelium mimicking the largely static or low flow conditions within the colonic lumen. A model inflamed colonic epithelium was generated by the addition of tumor necrosis factor-α (TNF-α) and lipopolysaccharide (LPS) to the basal and luminal epithelium sides, respectively. Co-culture of *A. hallii* with the LPS/TNF-α treated intestine diminished IL-8 secretion by ≥40% which could be mimicked by co-culture with the *A. hallii* metabolite butyrate. In contrast, co-culture of the inflamed epithelium with two strains of lactic acid-producing bacteria, *Lactobacillus rhamnosus GG* (*LGG*) and *Bifidobacterium adolescentis* (*B. adolescentis*), did not diminish epithelial IL-8 secretion. Co-culture with colonic epithelial cells from different donors demonstrated a consistent anti-inflammatory effect by *A. hallii*, but distinct responses to co-culture with *LGG* and *B. adolescentis*. The demonstrated system offers a simple and easily adopted platform for examining the physiologic impact of alterations in the intestinal epithelium that occur in the presence of probiotic bacteria and their metabolites.

## Introduction

Humans host hundreds of trillions of bacteria throughout the body. The colon or large intestine possesses the majority of these microbes having significant impact on the health and disease states of the host. Significant alterations in the gut microbe composition are linked to multiple diseases, including metabolic (Dabke et al., 2019), immune (Lazar et al., 2018), and neuronal (Kim et al., 2019; Muller et al., 2020) diseases. Additionally, the colonic bacterial composition modulates the efficacy of anti-cancer treatments (Ma et al., 2019) and vaccine response (Hagan et al., 2019) demonstrating the broad impact of these resident microbes on disease response by the host. In turn, essentially every aspect of a human’s makeup, for example, genetics (Goodrich et al., 2014; Goodrich et al., 2017), diet (Zmora et al., 2019), and aging (Wilmanski et al., 2021), directs the colonic microbial makeup. Diet is one of the most accessible and important ways to modulate the colonic microbiota and thus is of great interest in disease treatment and prevention. Generally higher dietary fiber intake is associated with higher colonic microbial diversity which in turn correlates with a healthier species profile and decreased disease burden (Makki et al., 2018). Given the broad health implications of the colonic microbes, much work is now underway to understand how colonic microbiota impact human health as well as to develop tailored microbiota interventions for disease prevention.

Ingestion of probiotics or bacterial supplements initially isolated from fecal samples of healthy individuals is a popular approach to attempt alteration of the colonic microbiota. Probiotics are formally defined as “live microorganisms that when administered in adequate amounts confer health benefit on the host (Hill et al., 2014).” Probiotic strains in commercially available probiotic supplements frequently include *Lactobacillus* and *Bifidobacterium* and are categorized as “generally recognized as safe (GRAS)” by health authorities. Many preclinical and clinical studies have demonstrated health benefits for some probiotics, for example, in the prevention and treatment of pathogenic infections by *Clostridium difficile* (Koretz, 2018; Suez et al., 2019). Reports also suggest that the supplements may diminish inflammatory bowel disease (IBD) and irritable bowel syndrome (IBS). However, other reports present contradictory evidence or even negative health effects (Suez et al., 2019). As a result, no formulation of probiotic bacterial strains has yet been approved for therapeutic or preventative use by the United States or European health authorities (Suez et al., 2019). Challenges in the methodology and/or data analysis of these preclinical and clinical studies may contribute to the disparate outcomes of probiotic studies (Koretz, 2018; Suez et al., 2019). But perhaps the biggest barrier to probiotic usage in medicine is that little is understood as to the mechanism by which these probiotic strains interact with host cells to impact health and disease.

Abundant information (genomics, proteomics, and metabolomics) as to the makeup of the human colonic microbiota is now available from the Human Microbiome Project (Human Microbiome Project Consortium, 2012a; Human Microbiome Project Consortium, 2012b; Integrative HMP Research Network Consortium, 2014) which is providing a path forward to discover probiotic strains. Several colonic bacterial strains have been suggested as “next-generation probiotics” based on predicted mechanistic links to potential health-promoting outcomes (El Hage et al., 2017; Lordan et al., 2020). Identified microbial metabolites can now be screened for their impact on cell physiology *in vitro* and linked to the microbes that produce the metabolites. For example, butyrate and other short chain fatty acids (produced by bacterial fermentation of fiber by *A. hallii* and other microbes) modulate inflammatory responses *in vitro* by altering secretion of anti-inflammatory cytokines of epithelial cells (Wang et al., 2018b) and white blood cells (Säemann et al., 2000; Nancey et al., 2002) to decrease inflammation (Chang et al., 2014; Wang et al., 2018b). However, despite ample evidence on the health-promoting effects of many microbial metabolites on human physiology, direct demonstration that the bacteria producing these metabolites also result in the same physiologic outcomes has proven difficult.

A major challenge in understanding the beneficial effects of probiotics has been the paucity of model systems that can simultaneously accommodate a primary human colonic epithelium, and the anaerobic probiotic bacteria for which O_2_ is toxic. A few studies have demonstrated successful co-culture of anaerobic bacteria and in particular butyrate-producing bacteria such as *Faecalibacterium prausnitzii*, *Eubacterium rectale*, or *Anaerobutyricum hallii* (*A. hallii*) with human primary colon epithelial cells (Zhang et al., 2021) or human colonic cancer cell lines (Shin et al., 2019). These studies all used flow-based co-culture systems which continuously flush out non-binding bacteria and endotoxins to minimize waste/toxin interactions with the epithelial cells. Given that a major function of the colon is to act as a waste storage organ for prolonged periods, these systems may not accurately mimic the largely static conditions of the human colon (Bassotti and Gaburri, 1988). Many studies also use tumor cells as surrogates for normal human intestinal epithelial cells, but for a multitude of reasons these tumor cells do not reflect normal physiology. For example, butyrate paradoxically increases IL-8 secretion in these tumor cells (Fusunyan et al., 1998) in contrast to the decreased IL-8 secretion of normal epithelium in the presence of butyrate (Wang et al., 2018b).

The goal of the current work was to design and optimize a platform for co-culture of normal human colonic epithelial cells with anaerobic probiotic bacteria found in the human colon. A cassette was developed to provide O_2_ to the basal side of primary human colonic epithelial cells while maintaining a very low O_2_ environment at the luminal side of the epithelium. Epithelial cell viability and differentiation were assessed under these culture conditions. The suitability of the luminal reservoir for growth of the O_2_ intolerant microbe, *A. hallii*, was optimized and the impact of the bacteria on the health of the epithelial cells was assessed. The intestinal epithelium modulates immune responses by secreting cytokines to recruit immune cells. IL-8 is the most abundant secreted cytokine and recruits neutrophils to the inflamed regions. Excessive or prolonged neutrophil infiltration into the epithelium compromises the epithelial integrity producing chronic inflammation (Chin and Parkos, 2007). Therefore, we investigated the modulation of epithelial cell inflammation by *A. hallii* by measuring the secretion of the neutrophil-recruiting pro-inflammatory cytokine IL-8 with and without tumor necrosis factor-α (TNF-α) and lipopolysaccharide (LPS). For comparison, two conventional probiotic strains, *L. rhamnosus GG* (*LGG*) and *Bifidobacterium adolescentis* (*B. adolescentis*) that produce lactic acid were co-cultured with colonic epithelium and their anti-inflammatory effect was assayed. Since humans possess considerable variability in their response to therapeutic interventions, epithelium from multiple human donors was also employed. This platform will be a powerful tool to investigate the impact of probiotic bacteria and their metabolites on the human colonic epithelium and more broadly on human health.

## Materials and Methods

### O_2_ Gradient Cassette

**FIGURE 1:**
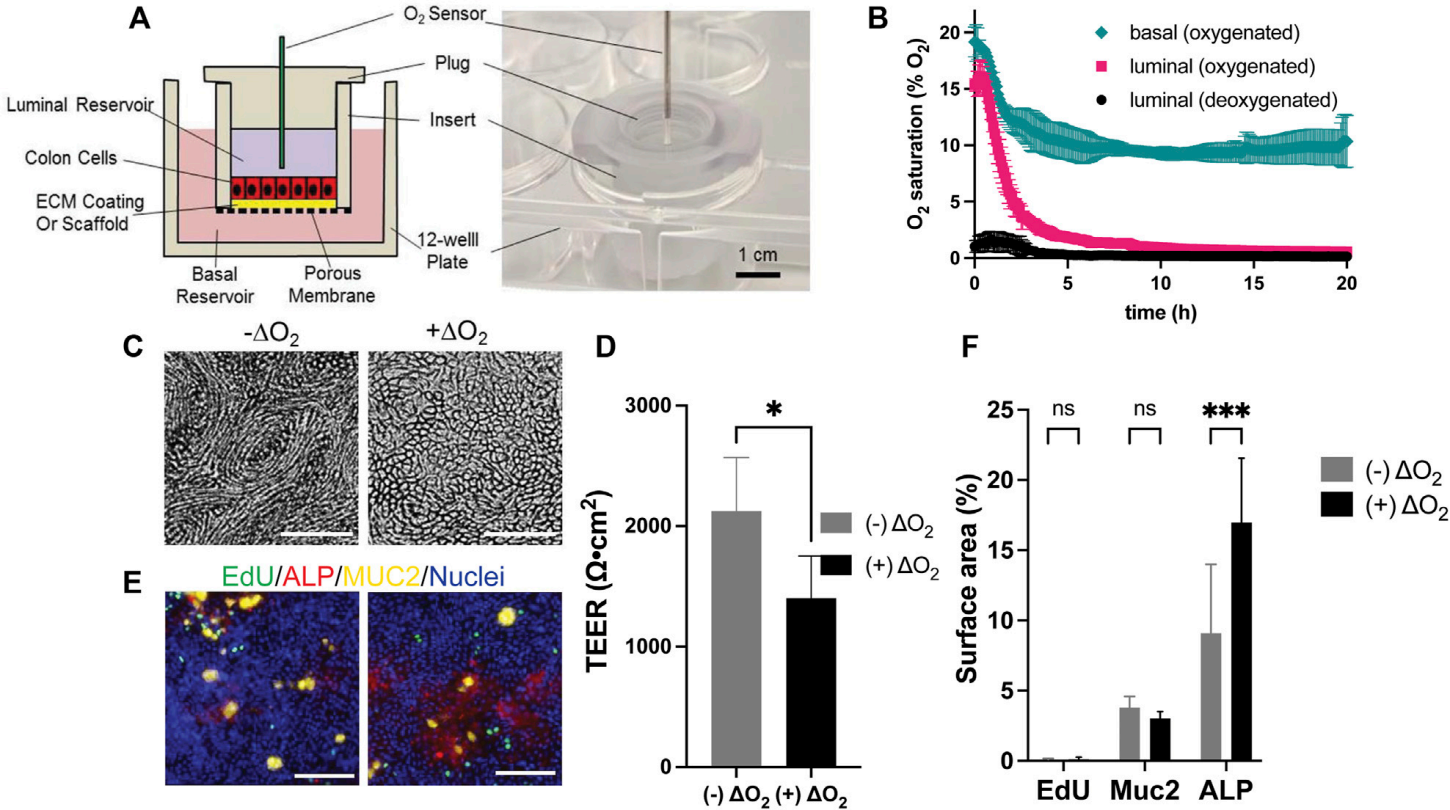
Design and culture of epithelial cells within the O_2_ gradient cassette. **(A)** Shown is a cross-sectional schematic (left panel) and a tilted top-view photograph (right panel) of the cassette with inserted luminal O_2_ sensor. **(B)** Measured O_2_ saturation over time in the luminal and basal reservoirs. Time zero was when the plug was installed. Shown is the luminal O_2_ saturation when deoxygenated (black circle) or oxygenated medium (magenta square) was loaded into the luminal reservoir. In both conditions the basal reservoir received oxygenated medium. Shown also is the O_2_ saturation (green diamond) in the basal reservoir when the luminal and basal reservoirs both received oxygenated media. The data points represent the average of the measurements, and the error bars the standard deviation of the data (*n* = 3 independent cultures). **(C)** Brightfield images of cells with and without an O_2_ gradient (ΔO_2_). **(D)** Measured TEER values of the epithelial cells with and without an O_2_ gradient (n = 4 technical replicates). **(E)** Confocal fluorescence images of the cells with (right) and without (left) an O_2_ gradient (ΔO_2_). EdU incorporation (green), MUC2 immunostaining (yellow), ALP activity (red), or Hoechst 33,342-stained DNA (blue). Scale bar = 100 µm. **(F)** The amount of EdU incorporation, MUC2 immunostaining, and ALP activity was quantified by plotting the percentage of the culture area positive for these stains divided by the surface area positive for Hoechst 33342 to normalize for the cell number. The data points represent the average of the measurements, and the error bars are the standard deviation of the technical replicates for n = 4 technical replicates. Unpaired *t*-test: *p ≤ 0.05; N.S. *p* > 0.05.

The O_2_ gradient cassette was comprised of a cell culture insert and a plug prepared as previously described (Kim et al., 2019) with modification. The cell culture inserts and the plugs were machined from polycarbonate to mate with a conventional 12-well plate (Figure 1A). After machining from polycarbonate, a porous PET membrane (0.4 µm pores, Sterlitech Cat #1300016) was attached to the insert base using medical grade, double-sided tape (3M, Cat #1504XL). Residual PET membrane overhanging the insert was trimmed with a surgical blade. Dust and debris on the inserts were removed using an air stream. Cell culture inserts and plugs could be reused multiple times after cleaning and disinfection. After each culture usage, the porous membranes were removed from the cell culture inserts as were the plugs. The cell culture inserts and the plugs were then washed in detergent (Decon Labs. Inc., Bacdown^®^Gel No-Rinse Skin Cleaner), sonicated, rinsed with deionized water, and autoclaved. For culture with an O_2_ gradient, a plug was threaded into a cell culture insert to create an O_2_-impermeable seal. A port in the plug was utilized for inoculating and sampling bacteria from the supernatant and sealed with EPDM rubber caps (McMaster-Carr, Cat #6448K117) when not in use.

### Extracellular Matrix Preparation

Prior to ECM coating, the cell culture inserts with porous membrane were treated with a plasma cleaner (Harrick Plasma, Cat #PDC-32G) for 5 min to clean the devices and facilitate adhesion of ECM molecules, disinfected with 70% ethanol, and air-dried in a biosafety cabinet. Then Matrigel (Corning, Cat #354234) was coated onto the plasma-treated PET membrane by incubation with 0.5 ml of 1% Matrigel (0.12 mg/ml at final concentration) in PBS for at least 30 min at 37°C. The cells were seeded immediately on the Matrigel layer.

### Human Colon Epithelial Cell Culture

Human colonic crypts isolated from transverse colons of a male and a female cadaveric donor [D1-male, RRID: CVCL_ZL23 (https://web.expasy.org/cellosaurus/CVCL_ZR41), D2-female, RRID: CVCL_ZR42 (https://web.expasy.org/cellosaurus/CVCL_ZR42)] were used. The colonic epithelial cells were expanded and routinely maintained on a collagen scaffold in a 6-well plate as described in the Supporting Information and previously reported (Wang et al., 2017). When the cells were seeded in the cell culture inserts, the cells from a single well of the 6-well plate were sub-cultured into 6 cell culture inserts pre-coated with Matrigel in the indicated medium (Supplementary Table S1). For measurement of the O_2_ concentration, viability, and cell phenotype under an O_2_ gradient, the cells were cultured in normoxic (i.e., aerobic) condition for 7 d and then further cultured for 3 d under the O_2_ gradient, i.e., with the plug installed into the cell culture inserts. The cells cultured under normoxic conditions were prepared in parallel in the same manner but without installation of the plug. For these experiments, the Matrigel-coated PET membrane was chosen (rather than a soft collagen substrate) since the stiffer surface promotes a fully differentiated, confluent monolayer with high TEER which more accurately reflects the cell type and monolayer features that interface with bacteria in the living human.

### Measurement of O_2_ Concentration and Transepithelial Electrical Resistance

The luminal and basal O_2_ concentrations were measured in separate experiments using a Microx 4 O_2_ sensor (PreSens, Germany) with a needle-type O_2_ probe (PreSens, Cat #NTH-PSt7). For luminal O_2_ concentration measurements, a needle-type O_2_ probe was located 1 mm above the cells by piercing through the rubber cap that seals the port of the plug. For the basal O_2_ level measurement, a custom-built reservoir with the same dimensions as that of a 12-well plate reservoir was fabricated with a hole enabling placement of the needle-type O_2_ probe near the bottom of the cell culture insert. O_2_ concentration measurement was performed at 37°C while pressure was recorded by the auxiliary pressure probe. The O_2_ level was measured and recorded every 5 min for at least 20 h for both luminal and basal reservoir O_2_ measurements. For TEER measurement, a volt-ohmmeter (World Precision Instrument, EVOM2) was used to measure TEER with a chopstick electrode. TEER was measured at 25°C immediately before and after the co-culture.

### Assessment of Cell Health and Phenotype

Measurement of S-phase cells was performed by incubating a thymidine analog 5-ethynyl-2′-deoxyuridine (EdU, Lumiprobe, Cat #10540, 10 μM) for 24 h with the cells. The cells were then washed and assayed for alkaline phosphatase (ALP) enzymatic activity by a staining kit (Vector Laboratories, Cat #SK-5100) following the manufacturer’s protocol. The cells were then immediately fixed with paraformaldehyde (PFA, 4%, 15 min, 25°C), permeabilized with 0.5% Triton-X 100 in PBS for 20 min at 25°C, then washed with 3% bovine serum albumin (BSA) in PBS. EdU incorporation into cellular DNA was detected by performing a click reaction with sulfo-cyanine5 azide (2 μg/ml, Lumiprobe Cat #A3330), CuSO_4_ (2 μM, catalyst) in the presence of sodium ascorbate (40 mg/ml) in PBS for 1 h at 25°C.

After performing the EdU incorporation, ALP activity assay, fixation and click reaction, the cells were blocked with 3% BSA for 1 h, and then incubated with mouse anti-mucin 2 (MUC2, Santa Cruz Biotechnology, Cat# sc-7314, RRID:AB_627,970) in 3% BSA at a 1:250 dilution for 16 h at 4°C. The samples were washed three times with 3% BSA in PBS and incubated with Alexa Fluor 488 conjugated goat anti-mouse secondary antibody (Jackson ImmunoResearch Labs, Cat# 115-545003, RRID:AB_2338840) at a 1:500 dilution in 3% BSA in PBS containing Hoechst 33342 (Thermo Fisher, Cat #H1399, 2 μg/ml) for 1 h at 25°C. The samples were washed once with 3% BSA and then with PBS.

Staining within the samples was then quantified by fluorescence microscopy using a laser scanning confocal microscope (Olympus, Fluoview 3000) with an excitation of 640 nm, 561 nm, 488 nm, and 405 nm and emission of 610–710 nm, 570–590 nm, 500–520 nm, 430–470 nm, respectively. ImageJ (https://imagej.nih.gov/ij/) was used to measure the fluorescence intensities of EdU, ALP, MUC2, and Hoechst 33342 stains as described previously (Wang et al., 2018a; Wang et al., 2018b; Kim et al., 2019). ImageJ was used to quantitatively measure the surface area possessing an empirically determined supra-threshold fluorescence for each of the stains. The coverage % is calculated from the percentage cell area positive for the EdU, ALP, or MUC2-positive area divided by Hoechst 33342-positive area.

### Bacterial Maintenance

*A. hallii* (ATCC27751, DSM 3353) was purchased from ATCC and propagated anaerobically using an anaerobic chamber (Coy Laboratory Products) and BD GasPak EZ Gas Generating System in peptone yeast glucose (PYG) medium supplemented with sodium acetate [PYG + A medium, peptone 10 g/L, yeast extract 10 g/L, dextrose 5 g/L, resazurin 1 mg/L, L-cysteine·HCl 0.5 g/L, CaCl_2_·H_2_O 100 mg/L, MgSO_4_ 7H_2_O 50 mg/L, K_2_PO_4_ 40 mg/L, KH_2_PO_4_ 40 mg/L, NaHCO_3_ 0.4 g/L, NaCl 80 mg/L, sodium acetate (5 g/L), hemin (5 mg/L) and vitamin K (1 mg/L)]. *A. hallii* were plated on PYG + A agar (PYG + A with 15 g/L agar), cultured in a plastic bag that contained BD GasPak EZ Gas Generating system sachet 37°C. One day prior to each experiment a fresh subculture was generated from a single colony for co-culture experiments by culturing in the broth for 16 h.

*L. rhamnosus GG* (*LGG*, ATCC 53103, purchased from Microbiologics, Cat #01090P) was propagated in a normoxic condition in De Man, Rogosa, Sharpe (MRS) medium (BD, Cat #288130) at 37°C. *LGG* were plated on MRS agar plate, and a single colony was used to prepare an overnight culture in MRS broth 16 h before co-culture experiments.

*Bifidobacterium adolescentis* (*B. adolescentis*, ATCC 15703) was purchased from ATCC and propagated anaerobically in PYG medium (the same composition as PYG + A above, but without sodium acetate) at 37°C. An overnight culture was prepared in PYG medium from a single colony formed on a PYG agar plated 16 h before the co-culture experiments.

### Bacterial Co-Culture Experiments

Matrigel-coated cell culture inserts were prepared as described above. The human colonic epithelial cells were resuspended in Medium 2 (Supplementary Table S1) and then seeded onto the Matrigel-coated inserts. The individual cell culture inserts were placed in a single well of a 12 well plate containing Medium 2 (2.5 ml per well) and incubated in a CO_2_ incubator at 37°C for 6 d. The culture medium was exchanged every other day. On day 6, the medium was removed and Medium 3 (Supplementary Table S1) and Medium 4 (which contained 10% L-WRN, a conditioned medium with Wnt-3a, R-spondin-3, noggin) without antibiotics were added to the luminal (0.25 ml) and basal (2.5 ml) reservoirs, respectively. The cells were cultured for 3 days with a media exchange on the second day. On day 9, the cells were moved to an anaerobic chamber (Coy Laboratory Products, 5% CO_2_, 95% N_2_), the luminal medium was replaced with deoxygenated fresh co-culture medium (0.6 ml) comprised of 10% PYG medium in Hank’s balanced salt solution (HBSS), and then the plug was installed into the cell culture insert. For bacterial co-culture, bacteria of interest were inoculated through the hole and the hole was sealed with a rubber cap. The cells were then brought out of the anaerobic chamber and the basal medium was replaced with fresh Medium 3 supplemented with 10% L-WRN (3 ml). For the LPS/TNF-α stimulation experiments, the cells were cultured the same way except that Medium 3 was used in both the luminal and basal compartments starting at day 6. On day 9, 10 ng/ml LPS from *Salmonella* (Sigma, Cat #L-6143) was added into the luminal co-culture medium and 10 ng/ml TNF-α in Medium 3 (Supplementary Table S1) was used for the basal medium during the co-culture. 5 × 10^4^ bacteria/ml was inoculated for all bacterial strains used. For butyrate and lactate treated samples (without bacteria exposure) the luminal co-culture medium with 3 mM sodium butyrate (Sigma, Cat #303410-500G) or 5 mM sodium DL-lactate (TCI, Cat #S0928) was added to the luminal compartment only without bacteria.

### Immunofluorescence for the Tight Junction Marker ZO-1

The cells were fixed by placement into prechilled methanol for 16 h, washed with PBS, and then blocked with 3% BSA in PBS for 1 h. Primary antibody directed against ZO-1 (Proteintech, Cat # 21773-1-AP, RRID:AB_10733242) was diluted in 3% BSA in PBS at 1:200 ratio and incubated with the cells for 16 h at 4°C. The cells were then washed 3 times with 3% BSA. A secondary antibody conjugated with Alexa Fluor 647 (Thermo Fisher Scientific, Cat # A27039, RRID:AB_2536100) was diluted at 1:500 ratio in 3% BSA in PBS and incubated with the cells for 1 h at 25°C. The cells were washed with 3% BSA in PBS, and then washed twice with PBS. The cells were examined by confocal fluorescence microscopy (Olympus, Fluoview 3000) with a 640 nm laser for excitation and emitted light collected (650–750 nm).

### Measurement of Cell Death

The membrane-impermeable DNA dyes, Sytox Green (Thermo Fisher, Cat #S7020) or propidium iodide (PI, Thermo Fisher, Cat #P3566), were used to label dead cells. Hoechst 33342 and/or Syto 9 (Thermo Fisher, Cat #S34854) was used to visualize the DNA in living human cells (Hoechst 33342, Syto 9) and bacteria (Syto 9) following the manufacturer’s protocols. For comparison of the cell viability in the normoxic environment to that under the O_2_ gradient without bacteria, the cells were washed with PBS, incubated with Sytox Green (1 μM) and Hoechst 33342 (10 μg/ml) at 37°C for 30 min and then imaged by confocal fluorescence microscopy.

Bacterial and human cell viability was assessed by adding PI (3.3 μg/ml) and Syto 9 (4.2 μM) to the luminal side of the O_2_ gradient cassette. Luminally delivered Syto 9 labeled nucleic acids in both the nuclei and cytoplasm of the human cells as well as that in the bacteria. Hoechst 33342 (10 μg/ml) was added into the basal side of the gradient cassette. Under these conditions, only DNAs in the human cell nuclei were labeled with Hoechst 33342. The cells were incubated at 37°C for 30 min. Then the cells were imaged confocally with × 4 objective, and 561 and 488 nm, 405 nm lasers for PI, Syto 9, and Hoechst 33342, respectively, for excitation, and emission was collected at 610–710 nm, 500–520 nm, and 430–470 nm, respectively. Five different locations (top, bottom, right, left, center) with imaging area of 10.12 mm^2^ at each location within the samples were imaged. The area of the images with a fluorescence intensity above an empirically set threshold was measured for PI, Syto 9, and Hoechst 33342 using Fiji software (Schindelin et al., 2012). The PI-positive area was divided by Syto 9 or Hoechst 33342-positive area and used as a metric for cell death.

### Measurement of Secreted IL-8

The IL-8 concentration in the basal media was assessed using an IL-8 ELISA kit (Thermo Fisher Scientific, Cat #88-8086-22) following the manufacturer’s protocol. The basal medium was diluted at 1:5 (without LPS/TNF-α) or 1:50 (with LPS/TNF-α) to obtain the measured signals within the dynamic range of the kit.

### Statistical Analyses

**FIGURE 2:**
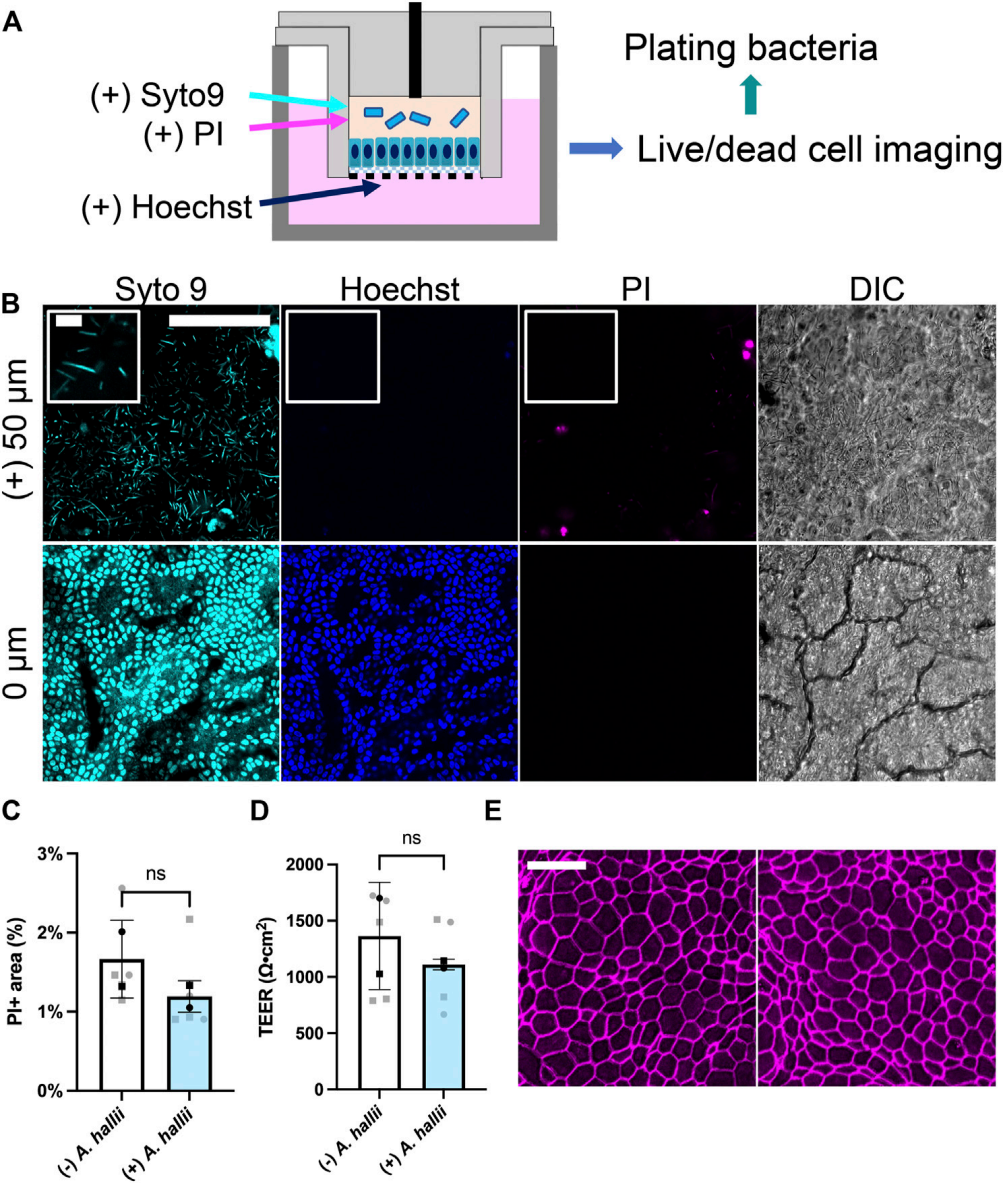
Co-culture of *A. hallii* with human colon epithelial cells in the O_2_ gradient cassette. **(A)** Schematic of the co-culture system. **(B)** Fluorescence confocal images were acquired after 24 h of co-culture at the plane of the colonic epithelium (0 μm) and 50 μm above the plane of the epithelial cell layer. Cyan: Syto 9, magenta: PI, blue: Hoechst 33342. Scale bar = 100 μm (10 μm in the insets). **(C)** Shown on the Y axis is the area of the culture positive for PI fluorescence divided by that positive for Hoechst 33342 fluorescence and **(D)** measured TEER. *n* = 5 data points from 2 biological replicates with 3 technical replicates of donor 1- square, two technical replicates of donor 2- circles. Black squares and black circles indicate the average of the replicates for donor 1 and 2, respectively. The bars represent the average of the biological replicates (donor 1 and 2), and the error bars the standard deviation of the data. Paired t-test was used for the statistical analyses. **(E)** Confocal fluorescence images of monolayers immunostained for ZO-1. Left: no bacterial exposure, right: *A. hallii* co-culture. Scale bar = 20 μm.

**FIGURE 3:**
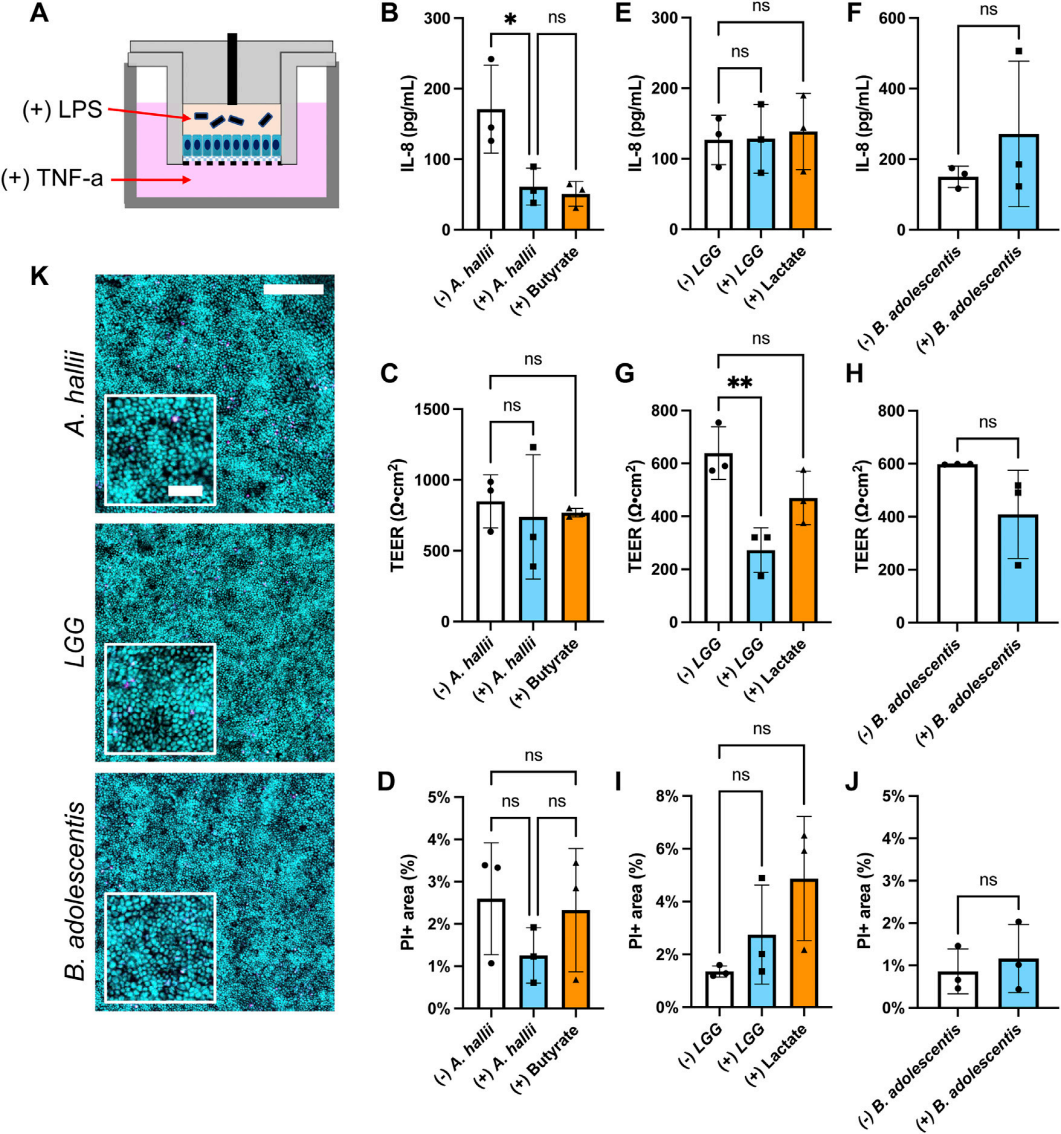
Impact of bacteria on IL-8 secretion by LPS/TNF-α treated intestinal epithelium. Donor 1 tissue was utilized for these experiments and 5 × 10^4^ CFU/ml of bacteria was inoculated onto each epithelial cell sample. **(A)** Schematic of the co-culture experiments with inflammatory stimulants **(B,C,E–H)** IL-8 concentration **(B,E,F)** and TEER **(C,G,H)** measured after 24 h culture with and without **(B,C)** *A. hallii*, **(E,G)** *LGG*, or **(F,H)** *B. adolescentis*. For (+) butyrate or (+) lactate samples, 3 mM butyrate **(B,C)** and 5 mM lactate **(C,G)** were added to the luminal media without addition of bacteria. The bars represent the average of the measurements, and the error bars indicate the standard deviation of the data. *n* = 3 technical replicates for all data. *p ≤ 0.05; **p < 0.01; ns. not significantly different **(D,I,J)** Epithelial cell death in the inflamed epithelial cell model after coculture with bacteria. Shown on the y-axis is the area positive for PI fluorescence divided by that positive for Hoechst 33342 fluorescence- PI area (%). The human colonic epithelial cells from donor 1 were cocultured with **(D)** *A. hallii*, **(I)** *LGG, or* **(J)** *B. adolescentis*. ns indicates not significantly different. *n* = 3 technical replicates for all data, except **(B)** control without bacterial exposure (*n* = 2). One-way ANOVA **(B-D,E,G,I)** and *t*-test **(F,H,J)** were used for the statistical analyses. **(K)** Fluorescence images of the human colon epithelial cells stained with Syto 9 (cyan) and propidium iodide (magenta) after co-culture with *A. hallii*, *LGG*, and *B. adolescentis* for 24 h. Scale bar = 200 μm in the larger image and 50 μm in the inset.

**FIGURE 4:**
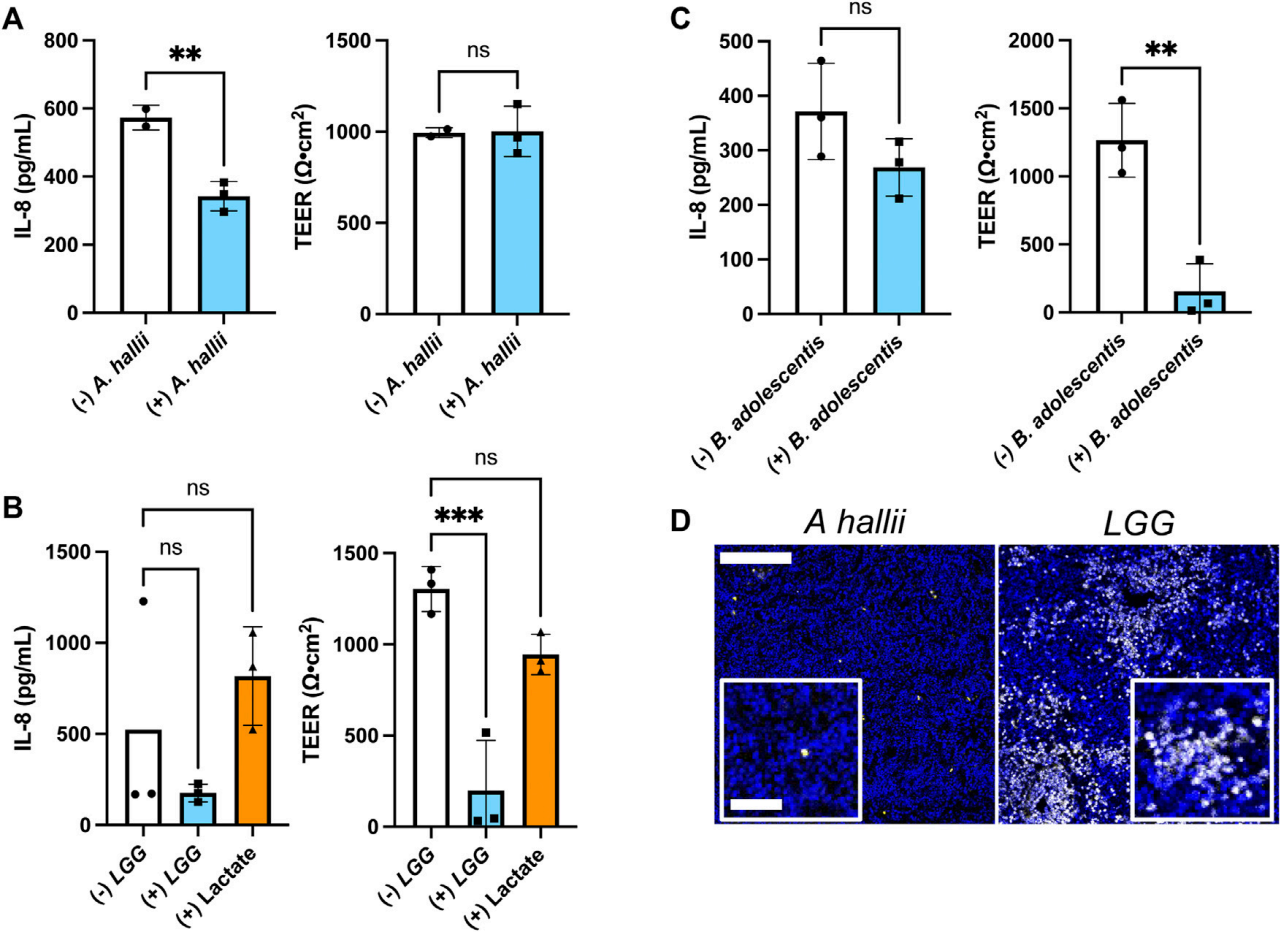
Impact of bacteria on IL-8 secretion by inflamed intestinal epithelium from donor 2 **(A–C)** IL-8 secretion and TEER measured after 24 h co-culture with and without **(A)** *A. hallii*, **(B)** *LGG*, or **(C)** *B. adolescentis*. 5 × 10^4^ CFU/mL of bacteria was inoculated onto each sample. The bars represent the average of the measurements, and the error bars indicate the standard deviation of the data. *n* = 3 technical replicates for all data. *p ≤ 0.05; **p < 0.01; ns. not significantly different. **(D)** Fluorescence of human colon epithelial cells stained with PI (yellow) and Hoechst 33,342 (blue) after co-culture with *A. hallii* and *LGG* for 24 h. Scale bar = 100 μm (50 μm in the insets).

GraphPad Prism 9 were used for statistical analyses of the data. An unpaired *t*-test was used to calculate *p* values for Figure 1, Figure 2, Figure 3D, Figures 4A,C; Supplementary Figure S1C, Supplementary Figures S2B,D, and ordinary one-way ANOVA for Figures 3B,C, Figure4B, Supplementary Figure S3A,B.

## Results and Discussion

### Design of a Cassette to Co-Culture Normal Human Colonic Epithelium and Probiotic Anaerobic Bacteria

A cassette was designed to mimic the luminal anaerobic and basal oxygenated microenvironments of the colonic epithelium while supporting the health and wellbeing of both primary human colonic epithelial cells and anaerobic bacteria for assay times up to 3 days. The design featured three major components: 1) a cell culture insert (hanging basket design) to support the formation of an epithelial cell monolayer and containing the luminal medium for bacterial growth, 2) a plug that covers the cell culture insert to block the O_2_ influx into the luminal side of the cells, and 3) a basal reservoir to store the oxygenated basal cell culture medium (Figure 1A). A key goal of the cell culture insert was to support the formation of a high resistance epithelial cell monolayer blocking passive movement of cytokines and microbes between the luminal and basal compartments. To achieve this goal as well as to provide high-quality imaging of both epithelial and bacterial cells, the luminal insert surface was covered with a thin layer of extracellular matrix distinguishing this design from a precursor which employed a thick collagen scaffold (Kim et al., 2019). An O_2_-impermeable, polycarbonate plug mated to the culture insert, incorporating an O_2_ sensor port for monitoring the luminal O_2_ saturation since the anaerobic bacteria colonizing the intestine require <2% O_2_ (Sheridan et al., 1990). The port also supported the introduction and removal of bacteria during the experiments. The basal reservoir was formed from a standard multiwell plate which exposed the basal fluid compartment to the atmosphere with a goal of maintaining a high O_2_ saturation (>10% O_2_) in the basal medium and creating a steep O_2_ gradient between the luminal and basal compartments. Finally, the use of the hanging basket cell insert enabled a static epithelial-bacterial cell co-culture matching the low-flow or storage conditions of the human colon and providing a platform matching that in current use by biomedical labs with a goal of encouraging device adoption by the life science community.

### Measurement of O_2_ Saturation

Human colonic epithelial cells consume significant quantities of O_2_. Prior work has demonstrated that this consumption is sufficient to generate an anaerobic environment within a low volume compartment surrounded by O_2_ impermeable walls (Kim et al., 2019). Thus, it was expected that installation of the plug into the cell culture insert in the presence of a confluent epithelial monolayer would prevent O_2_ replenishment in the luminal reservoir once the cells consumed available O_2_. An anaerobic microenvironment would then be created in the luminal reservoir. In contrast, the liquid in the basal reservoir remains in contact with the atmosphere so that O_2_ can diffuse into the liquid as it is consumed by the cells. For this reason, the fluid in the basal compartment was expected to remain oxygenated. Initially both the luminal and basal reservoirs were filled with medium that was normoxic (∼21% O_2_), i.e., in equilibrium with the atmosphere. The luminal O_2_ saturation decreased with an initial average rate of 3.5% O_2_/h reaching 2% O_2_ by 4.3 h demonstrating that the epithelial cells were competent to deplete O_2_ from the overlying medium (Figure 1B). The O_2_ saturation in the basal compartment diminished at an average initial rate of 1.74% O_2_/h stabilizing at 10% at 5 h suggesting that O_2_ readily diffused into the basal medium from the openings around the insert. When deoxygenated medium (0.2% O_2_) was placed at time zero into the luminal reservoir, the O_2_ saturation of the luminal medium remained below 2% O_2_ at all times and after an initial increase returned to 0.2% O_2_ by 3.75 h. The basal O_2_ saturation in the presence of the deoxygenated luminal medium was not significantly different from that when normoxic medium was initially placed into the luminal compartment. Given the high metabolic demands of the colonic epithelium, the basal medium was changed every 2 d and during these medium exchanges, the luminal O_2_ always remained less than 0.8% O_2_ at every tested time point (Supplementary Figure S1), demonstrating the robustness of forming and maintaining an O_2_ gradient in the device.

### Measurement of Epithelial Cell Properties Under an O_2_ Gradient

Human colonic epithelial cells from a single donor (donor 1) were cultured for 7 d in normoxic condition to form continuous, high-TEER cell monolayers with TEER = 1819 ± 195 Ω⋅cm^2^ (*n* = 12 samples). Then the monolayers were cultured under the O_2_ gradient for 3 d and their viability, proliferative capacity, and differentiated-cell lineage allocation were compared to that of cells grown under normoxic conditions. When viewed under brightfield, an O_2_ gradient significantly changed the cell morphology (Figure 1C). Cells were relatively elongated and formed a vortex pattern in the absence of an O_2_ gradient. Under the O_2_ gradient the vortex pattern was lost, and the cells had the typical cobblestone appearance of colonic epithelium. Cells cultured under the O_2_ gradient were also high in viability (99.93 ± 0.03%, *n* = 6) not significantly different from that of cells cultured in the absence of the gradient (99.25 ± 0.25%) (Supplementary Figure S2B). The TEER was high in both culture formats (O_2_ gradient 1,404 ± 349 Ω⋅cm^2^; normal O_2_ 2,126 ± 448 Ω⋅cm^2^, *n* = 4) suggesting that the epithelial cells tolerated the O_2_ gradient and formed a contiguous monolayer (Figure 1D). The percentage of cells in S phase indicative of a stem/proliferative cell compartment was low (Figures 1E,F) and not significantly different for the two culture systems. This is consistent with other reports that confluent monolayers under spontaneous differentiation and demonstrate decreased cell proliferation likely due to contact inhibition (Eagle and Levine, 1967). This result is distinct from that found when cells are in contact with very soft substrates (100 Pa) and it is likely that the O_2_ level works in concert with surface cues to direct cell outcomes. (Kim et al., 2019). The propensity of the cells cultured under the O_2_ gradient to differentiate into two major colonic cell types (absorptive colonocytes or mucus-secreting goblet cells) was next assessed. The density of MUC2 + goblet cells was not significantly altered between the O_2_ gradient and normoxic culture systems (Figures 1E,F). Significantly increased alkaline phosphatase activity was present under the O_2_ gradient relative to that under constant O_2_ conditions suggesting that the low luminal O_2_ tension might promote the formation of mature colonocytes (Figures 1E,F). Overall, these data confirm that the human colonic epithelial cells under a physiological O_2_ gradient maintain high cell viability and barrier integrity with the expected cellular phenotypes.

### Co-Culture of Colonic Epithelial Cells With *A. hallii*

*A. hallii* (Holdeman and Moore, 1974), also known as *Eubacterium hallii* (Shetty et al., 2018), is a gram-positive, obligate anaerobic strain belonging to the *Firmicutes* phylum and a normal resident of the human colon. As a potent butyrate producer, *A. hallii* has been categorized as a potential probiotic strain since its major metabolite butyrate possesses beneficial health effects, for example, a decreased the risk of colon cancer (Fekry et al., 2016). *A. hallii* was inoculated into the luminal reservoir of a cassette with human colonic epithelial cells grown for 1 d under the O_2_ gradient. (Figure 2A). After 24 h of co-culture, the number of viable bacteria increased 10-fold relative to that of the initial inoculum [from log (CFU/mL) = 5.36 ± 0.55 att = 0 to 6.70 ± 0.98 att = 1 d, *p* = 0.0278, *n* = 5, unpaired *t*-test, Supplementary Figure S2A], confirming that the anaerobic luminal environment supported growth of this obligate anaerobe. To image living bacteria adjacent to the colonic epithelium, the nucleic acid-binding dyes Syto 9 (membrane permeable, labeling all cells) and propidium iodide (PI, membrane impermeable, labeling dead cells) were added to the luminal reservoir staining nucleic acids in both the bacterial and human cells. The DNA-binding, membrane-permeable dye Hoechst 33342 was added to the basal medium and selectively labeled the colonic epithelial cells. When viewed by fluorescence microscopy, immotile, rod-shaped bacteria labeled with Syto 9, but not PI, were observed in the co-culture consistent with the known characteristics of viable *A. hallii* (Figure 2B), features that the cultures without bacterial exposure did not possess (Supplementary Figure S2B). Most bacteria were present above the plane of the epithelial cells suggesting that they did not bind tightly to the human cells. A monolayer of human epithelial cells was readily visualized by DIC microscopy and exhibited a cell morphology and appearance similar to that of epithelial cells in the absence of the bacteria (Figure 2B). The epithelial cells were stained with both Syto 9 and Hoechst 33342 but demonstrated very little PI uptake suggesting high viability.

The impact of *A. hallii* co-culture on the human colonic epithelial cells was assessed after 24 h by comparing PI and Hoechst 33342. Cell viability was high with *A. hallii* co-culture and was not significantly different from that without *A. hallii* (Figure 2C). TEER, a measure of epithelial barrier integrity of the human colon epithelial cells was not significantly altered by *A. hallii* co-culture (1,297 ± 464.7 Ω⋅cm^2^ without *A. hallii*, 1,118 ± 381.1 Ω⋅cm^2^ with *A. hallii*, Figure 2D). Similarly, immunofluorescence measurement of the tight junction marker ZO-1 (Figure 2E) demonstrated intact cell-cell junctions and with no obvious differences with and without *A. hallii* co-culture. Taken together, these data suggest that *A. hallii* co-culture did not compromise the barrier integrity of the human epithelial cell layer.

### Effect of Butyrate and Lactic Acid Producing Bacteria on the Colonic Epithelium

The anti-inflammatory impact of *A. hallii* on the colonic epithelium was assessed by measuring the amount of pro-inflammatory cytokine IL-8 secreted from inflamed human primary colonic epithelial cells. The epithelium from donor 1 was treated by addition of lipopolysaccharide (LPS) to the luminal reservoir and TNF-α to the basal reservoir of the human epithelial cells (Figure 3A). LPS is an endotoxin derived from the cell wall of gram-negative bacteria while TNF-α is an inflammatory cytokine secreted by mononuclear phagocytes including monocytes and macrophages (Friedrich et al., 2019). Compared to the control epithelium without bacteria, epithelium co-cultured with *A. hallii* exhibited a 65% decreased IL-8 secretion (Figure 3B). Butyrate (3 mM) added to the luminal medium of epithelial cells in the absence of *A. hallii* also decreased IL-8 secretion to a similar level, suggesting that production of butyrate by *A. hallii* might be in part due to this effect. All LPS/TNF-α treated epithelial cultures exhibited a similar TEER (Figure 3C) and cell viability (Figures 3D,K) suggesting that alteration of the monolayer barrier integrity was not an explanation for the diminished IL-8 production of the *A. hallii* or butyrate-exposed cells. These results demonstrate the anti-inflammatory impact of the butyrate-producing bacteria *A. hallii*. Notably without these inflammatory stimuli, *A. hallii* did not decrease IL-8 secretion suggesting that *A. hallii* exerts its effect primarily in the presence of inflammation (Supplementary Figure S2C). Interestingly in the absence of LPS/TNF-α, the concentration of IL-8 secreted to the basal side was greater than in the presence of LPS/TNF-α without bacterial exposure. This may be due to an accelerated rate of degradation of IL-8 in the presence of LPS and TNF-α. Alternatively, this may be an impact of the O_2_ gradient and reduced oxygen available to the cells or the presence (Figure 3) and absence (Supplementary Figure S2C) of Wnt, R-spondin, and Noggin in the culture medium. In these conditions, other cytokines including MCP-1 and IL-10 secreted to the basal medium were below the detection limit, which is consistent with prior cytokine secretion measurements (Wang et al., 2018b).

While *A. hallii* secretes butyric acid, other probiotic bacteria secrete lactic acid which may also act to decrease colonic inflammation by the same or a different mechanism. Two lactic acid bacterial strains, *LGG* and *B. adolescentis*, were assessed for their ability to modulate IL-8 secretion by LPS/TNF-α treated colonic epithelium. *LGG*, a gram-positive facultative anaerobic strain, is one of the most studied *Lactobacillus* strains for its perceived health promoting effect (Segers and Lebeer, 2014). *B. adolescentis* is a gram-positive obligate anaerobic commensal strain that is associated with good health (Arboleya et al., 2016). Each strain was co-cultured with LPS/TNF-α treated human colonic epithelial cells in the O_2_ gradient device for 24 h followed by measurement of IL-8 secretion. No statistical difference in secreted IL-8 was present with and without *LGG* or *B. adolescentis* co-culture (Figures 3E,F). Similarly, addition of lactate (5 mM) to the luminal compartment did not significantly alter IL-8 secretion under these conditions relative to the control (Figure 3E). Notably the TEER of the epithelial monolayer was significantly reduced in the presence of *LGG*, but not *B. adolescentis* or lactate (Figures 3G,H). However, cell death in the cultures with *LGG*, *B. adolescentis* or lactate was not significantly different from that of LPS/TNF-α treated epithelium alone (Figures 3I–K). Under these conditions, lactic acid-producing bacteria did not modulate IL-8 secretion and thus their probiotic effects may be due to other mechanisms (Hütt et al., 2006; Fan et al., 2021). These data suggest that this easy-to-perform *in vitro* co-culture model could provide a useful platform for assaying anti-inflammatory interactions of gut bacteria on human colonic epithelial cells.

### Donor Variability in the Response to Butyrate and Lactic Acid-Producing Bacteria

Dietary, microbial, and genetic differences create significant variability among individuals with respect to disease propensity, drug responses, and probiotic efficacy (Healey et al., 2017; Zmora et al., 2018). Thus, the colonic epithelium from a second donor (donor 2) was co-cultured with *A. hallii*, *LGG*, and *B. adolescentis*, and the impact of these anaerobic microbes on LPS/TNF-α tissue was assessed. As with donor 1, *A. hallii* co-culture significantly diminished IL-8 secretion by the epithelium derived from donor 2 with respect to controls without *A. hallii* (Figure 4A). Similarly, *A. hallii* co-culture did not alter barrier integrity (TEER) of the donor 2 epithelial monolayers. Co-culture of the LPS/TNF-α treated donor 2 epithelium with *LGG* or *B. adolescentis* did not significantly reduce IL-8 secretion relative to control epithelium as was observed with the donor 1 cells (Figures 4B,C; Supplementary Figure S3B). In contrast to donor 1 tissue, the TEER and viability of donor 2 tissue was significantly decreased by co-culture with the lactic acid-producing bacteria (Figures 4C,D; Supplementary Figure S3B), with 28 ± 19% of PI + or dead cells in the *LGG* coculture compared to 0.86 ± 0.21% in the *A. hallii* coculture (Figure 4D; Supplementary Figure S3A). This human-to-human variability may partially explain the inconsistent outcome of clinical trials assessing the probiotic impact of lactic acid-producing bacteria in humans (EFSA Panel on Dietetic Products and Allergies, 2011). It is clear that a much greater number of donors will be needed to assess the true variability of the human intestinal epithelium to the different microbes. Nevertheless, the demonstrated platform opens the door to providing large scale assessment of donor tissue responses to intestinal bacteria.

Under these conditions, LPS/TNF-α treatment of the epithelial cells did not induce significant IL-8 secretion (Figures 3, 4). It is possible that the unique O_2_ environment with luminal hypoxia in the O_2_ gradient may alter cytokine secretion by LPS/TNF-α. Additionally, medium components may alter cytokine secretion profiles. For example, antioxidants in the typical primary cell culture medium suppress cytokine responsiveness (Beaurivage et al., 2020; Ruan et al., 2020). Although many antioxidants such as N-acetylcysteine and B27 were removed from the culture medium, glutathione remained as a component in the Advanced DMEM base medium, which may have a suppressive effect on the cytokine secretion. FBS is another possible modulator that may reduce cytokine responsiveness to inflammatory stimuli (Antypas et al., 2014; Kumar et al., 2016). Nevertheless, co-culturing *A. hallii* consistently reduced the IL-8 secretion in cells from different donors compared to the control cells without bacterial exposure, demonstrating the anti-inflammatory effect of butyrate-producing bacteria.

## Conclusion

A simple platform was developed to enable the co-culture of probiotic, O_2_ intolerant microbes with an oxygenated human colonic epithelium. A self-sustaining O_2_ gradient was formed across a high-resistance, impermeable epithelial cell monolayer such that the medium on its basal side was rich in O_2_ while the medium on the luminal side was O_2_ depleted. This created a compartment mimicking the microenvironment of the colonic lumen and in which obligate anaerobic bacteria were able to thrive. The human colon epithelial cells growing under the O_2_ gradient differentiated into the two major intestinal cell types (goblet cells and colonocytes) without loss of cell viability or barrier integrity despite the adjacent microbes. Importantly, the platform includes a static, no-flow luminal compartment to replicate the conditions of the human colon which acts as a storage organ for fecal material with embedded microbes. *A. hallii*, a butyrate-producing bacteria and a potential candidate for the next generation of probiotics, grew robustly in the presence of the human primary colon epithelial cells without compromising epithelial cell viability or monolayer barrier function. Moreover, co-culture with *A. hallii* for 24 h significantly lowered secretion of the pro-inflammatory cytokine IL-8 by LPS/TNF-α treated epithelial cells from two different donors suggesting that a beneficial effect of *A. hallii* in the human gut might be a decrease in inflammation. Application of butyrate, a major metabolite of *A. hallii*, to the LPS/TNF-α treated epithelial cell monolayer mimicked the anti-inflammatory impact of *A. hallii*. Thus, butyrate production is likely to be one explanation for the beneficial effects of this microbe. In contrast, two strains of lactic acid-producing probiotic bacteria *LGG* and *B. adolescentis* did not lower epithelial-cell IL-8 secretion under the same conditions. Furthermore, the human primary colonic epithelial cells possessed limited tolerance to static co-culture with these anaerobic bacteria as evidenced by the decreased TEER and increased intestinal cell death. It should be noted that a thick mucus layer was absent in the current model although goblet cells were present. The absence of a mucus barrier could significantly alter the epithelial tolerance to the gut bacteria. These data also demonstrate the challenges of working with primary intestinal cell cultures in the presence of not only large numbers of bacteria but also with the O_2_-dependent epithelial cells immediately adjacent to a static anoxic medium to truly mimic the *in vivo* colonic conditions. Challenges in limiting bacterial growth consistently across experiments can occur due to varying compositions of biological reagents such as fetal calf serum, Matrigel, and the conditioned medium supplying Wnt-3a, R-spondin-3, and noggin. It is also increasingly clear that primary cell passage number is an important variable in all experiments when utilizing these donor-acquired cells. Finally, donor-to-donor variability in primary tissue responsiveness to stimuli can be quite significant. It will be important for future experiments to be performed in a significantly scaled-up manner with much larger numbers of replicates and donors to validate these initial feasibility studies. A thick mucus layer of several hundred microns will be a next step in model improvement. Nevertheless, the demonstrated *in vitro* model system with a physiological O2 gradient and static co-culture offers a valuable platform for initial screening efforts on the impact of probiotic bacteria on host physiologic responses.

## Data Availability

The raw data supporting the conclusion of this article will be made available by the authors, without undue reservation.
