# Supplementary material for: A Platform for Co-Culture of Primary Human Colonic Epithelium With Anaerobic Probiotic Bacteria
Source: Front Bioeng Biotechnol. 2022 Jun 8;10:890396. doi: 10.3389/fbioe.2022.890396 (PMC9213686; doi:10.3389/fbioe.2022.890396)
Supplement: Supplementary file 1 [file DataSheet1.docx]

Supplementary Material

# Supplementary Figures and Tables

## Supplementary Tables

## Supplementary Table S1. Media compositions used in this study.

|  | Maintenance medium (MM) | Medium 1 | Medium 2 | Medium 3 | Medium 4 |
| --- | --- | --- | --- | --- | --- |
| Advanced DMEM/F12  (Thermo Fisher) | 50% (v/v) | 50% (v/v) | 50% (v/v) | 90% (v/v) | 80% (v/v) |
| L-WRN conditioned medium* | 50% (v/v) | 50% (v/v) | 50% (v/v) | - | 10% (v/v) |
| GlutaMax  (Thermo Fisher) | 1x | 1x | 1x | 1x | 1x |
| HEPES | 10 mM | 10 mM | 10 mM | 10 mM | 10 mM |
| Human EGF or mouse EGF (for Medium 2 only) (Peptrotech) | 50 ng/mL | 50 ng/mL | 50 ng/mL | 50 ng/mL | 50 ng/mL |
| B27* | 1x | 1x | 1x | - | - |
| N-acetyl cysteine  (MP bio) | 1.25 mM | 1.25 mM | 1.25 mM | - | - |
| Gastrin  (Anaspec) | 10 nM | 10 nM | 10 nM | - | - |
| Y-27632  (ApexBio) | 10 μM | 10 μM | 10 μM | - | - |
| A83-01  (Sigma Aldrich) | 500 nM | - | - | 500 nM | 500 nM |
| Prostaglandin E2 (PGE2)  (Cayman chemicals) | - | - | 10 nM | - | - |
| Nicotinamide  (Sigma Aldrich) | - | - | 10 mM | - | - |
| SB202190  (Selleckchem) | 3 μM | 3 μM | 3 μM | - | - |
| Primocin  (InvivoGen) | 50 μg/mL | 50 μg/mL | 50 μg/mL | - | - |
| FBS  (HyClone, heat inactivated) | - | - | - | 10% (v/v) | 10% (v/v) |

## Supplementary Figures


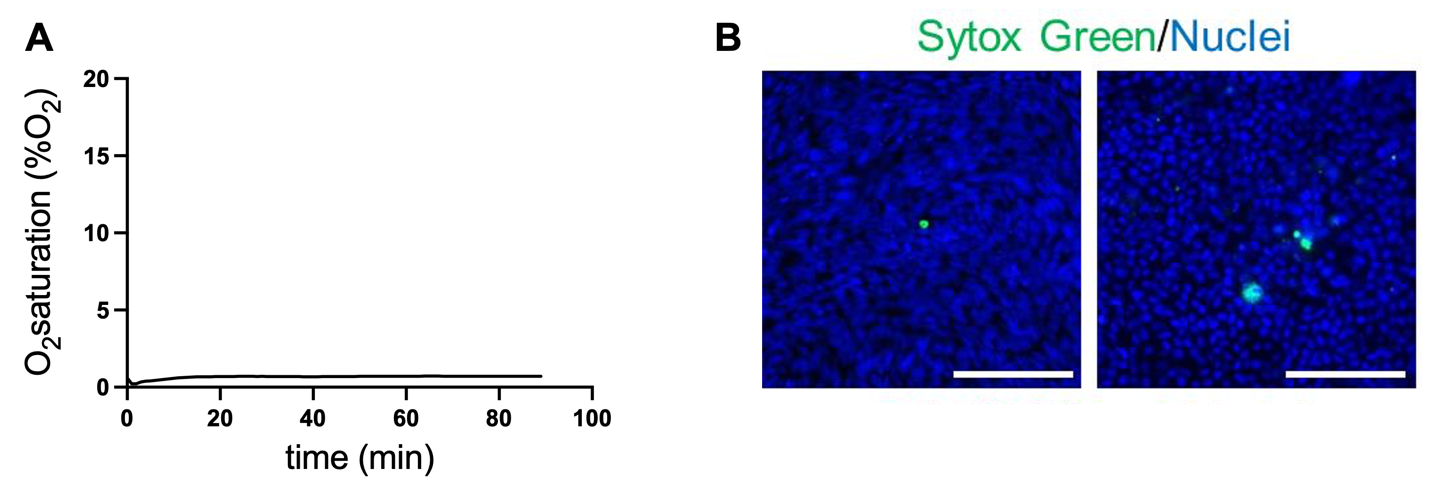


**Supplementary Figure 1.** O_2_ gradient cassette properties and its impact on colonic epithelial cells. (a) A typical example of the perturbation to the luminal O_2_ saturation when the basal medium was replaced with oxygenated medium (at t = 0). (b) Images of the human colonic epithelial cells stained with Sytox Green (green) and Hoechst 33342 (blue) with (left) and without (right) an O_2_ gradient. Scale bar = 100 µm.


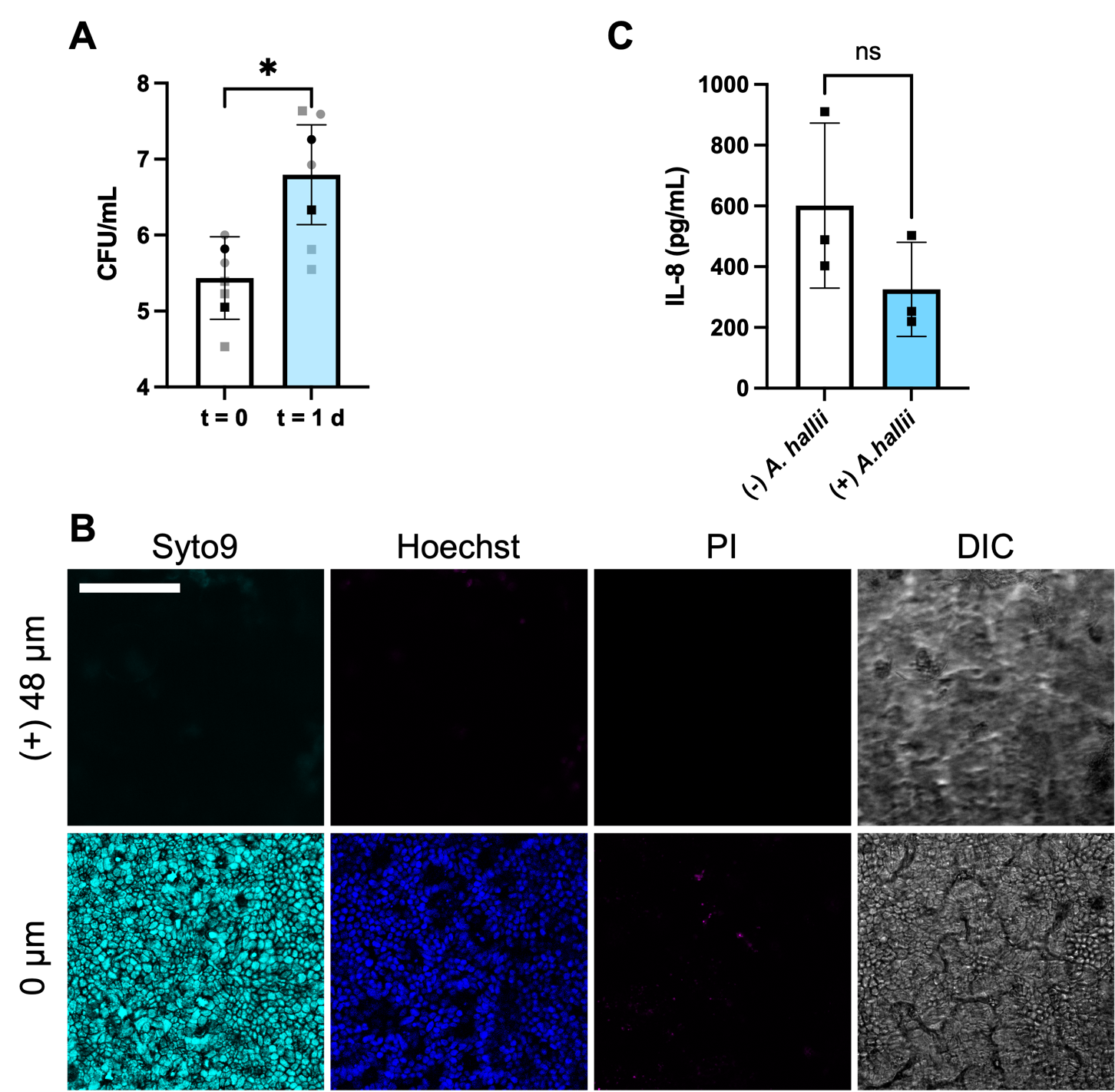


**Supplementary Figure 2.** Coculture of *A. hallii* and epithelial cells. (a) Colony forming units (CFU) of *A. hallii* added to the epithelial cells at t = 0 and present after 1 day of coculture. The bars represent the average of the measurements, and the error bars the standard deviation of the data. n=5 data points from 2 biological replicates with 3 technical replicates of donor 1- square, two technical replicates of donor 2- circles. Black squares and black circles indicate the average of the replicates for donor 1 and 2, respectively. Paired t-test was used. * p ≤ 0.05. (b) Fluorescent confocal images of the cells without bacterial exposure. Cyan: Syto 9, magenta: PI, blue: Hoechst 33342. “(+) 48 μm” indicates that the images were acquired at a focal plane 48 μm above the plane of the intestinal epithelium which was located at 0 μm. Scale bar = 100 μm. (c) IL-8 secreted into the basal compartment after 1 d of culture with and without *A. hallii*. The bars indicate the mean of the measurements, and the error bars the standard deviation of the data. n = 3 technical replicates from donor 1. ns indicates not significantly different.


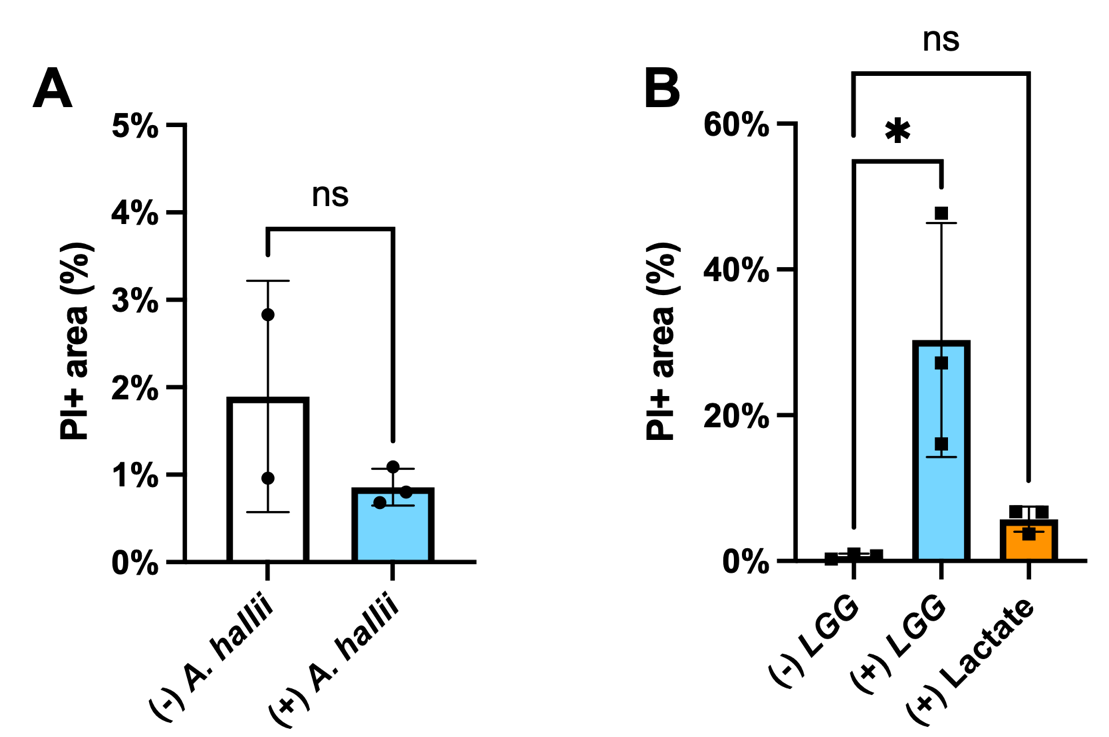


**Supplementary Figure 3.** (a,b) Epithelial cell death in the LPS/TNF-α treated epithelial cell model after coculture with (a) *A. hallii* and (b) *LGG*. The Y axis is the area of the culture positive for PI fluorescence divided by that positive for Hoechst 33342 fluorescence [PI area (%)]. The human colonic epithelial cells from the donor 2 were cocultured with (a) *A. hallii*, or (b) *LGG*. ns indicates not significantly different. n=3 technical replicates for all data, except (a) control without bacterial exposure (n=2). T-test (a) and One way ANOVA (b) were used for the statistical analyses.

# Supplementary Methods

## Culture of the human primary colon epithelial cells

The colonic epithelial cells were isolated from cadaveric donors (D1- male, RRID: CVCL_ZL23 (https://web.expasy.org/cellosaurus/CVCL_ZR41), D2 - female, RRID: CVCL_ZR42 (https://web.expasy.org/cellosaurus/CVCL_ZR42)) and cultured on soft collagen gel as previously described in detail.(Hinman et al., 2021) To prepare the collagen gel, 1 mg/mL of neutralized collagen solution was first prepared by neutralizing the collagen solution in 0.02 M acetic acid (354236, Corning) with a neutralization buffer containing NaOH, NaHCO_3_, HEPES in PBS (8.6 μM, 76 μM, 29 μM respectively at the final concentration in the collagen solution). Then 1 mL of neutralized collagen gel was placed in one 6-well plate and incubated for 1 h at 37°C to obtain a soft collagen gel. The cells were plated in the collagen gel in Maintenance Medium (Table S1) and subcultured every 5-7 days at a 1:3 ratio (from one 6-well to three 6-wells). For passaging, collagenase was used to degrade the collagen gel underneath the cells. Then the cells were further dissociated by incubating in 0.5 mM ethylenediaminetetraacetic acid (EDTA) in PBS for 5 min at 37°C followed by repeated pipetting. The cells were karyotyped at passage 7 and 15 to confirm the absence of chromosomal abnormality. The cells were used up to passage 15 for experiments.

## Quantification of viable bacteria in the co-culture

The number of viable bacteria in *A. hallii* co-cultures was estimated by counting the colony forming units (CFU) in the supernatants by streaking serial dilutions onto a PYG+A agar plate (the composition is described in the Materials and Methods section 2.6). For the CFU/mL measurement at t = 0, 5 μL of the coculture medium immediately after inoculation was sampled and subjected to plating on an agar plate for estimating CFU. After 24 h co-culture, the supernatant was collected and plated on agar plates after serial dilution. The colonies appeared on the agar plates and were counted after 24 h of anaerobic incubation at 37°C.

# References

Hinman, S.S., Wang, Y., Kim, R., and Allbritton, N.L. (2021). In vitro generation of self-renewing human intestinal epithelia over planar and shaped collagen hydrogels. *Nature Protocols* 16(1)**,** 352-382. doi: 10.1038/s41596-020-00419-8.
